# Effects of Resistant Starch on Patients with Chronic Kidney Disease: A Systematic Review and Meta-Analysis

**DOI:** 10.1155/2022/1861009

**Published:** 2022-07-18

**Authors:** Xinyi Du, Jing Wu, Chenlin Gao, Qinqin Tan, Yong Xu

**Affiliations:** ^1^Department of Endocrinology and Metabolism, The Affiliated Hospital of Southwest Medical University, Luzhou, Sichuan, China 646000; ^2^Cardiovascular and Metabolic Diseases Key Laboratory of Luzhou, Affiliated Hospital of Southwest Medical University, Luzhou, Sichuan, China; ^3^Sichuan Clinical Research Center for Nephropathy, Affiliated Hospital of Southwest Medical University, Luzhou, Sichuan, China

## Abstract

**Background:**

Chronic kidney disease (CKD) is a main health problem associated with increased risk of cardiovascular disease, morbidity, and mortality. Recent studies shown that the progression of CKD may be related to the change of intestinal flora. Resistant starch (RS) is a type of dietary fiber that can act as a substrate for microbial fermentation. Some studies have found that the supplementation of RS can improve the intestinal flora disorder in CKD patients. However, the specific effect of RS on CKD patients remains controversial.

**Objective:**

We designed this meta-analysis to identify and assess the effects of RS on patients with CKD.

**Methods:**

A comprehensive search of MEDLINE, Embase, Web of Science, and Cochrane systematic review databases was conducted in January 2020, and all new trials were updated in August 2021. Randomized trials were collected to assess the effects of RS on patients with CKD. The weighted average effect size of the net change was calculated by using the random-effects model.

**Results:**

The meta-analysis included 8 studies involving 301 participants. RS intake significantly reduced serum indolephenol sulfate (IS), blood phosphorus, IL-6, and uric acid levels in dialysis patients. The mean difference (MD) of serum IS (*P* = 0.0002) in the dialysis subgroup was -12.57 *μ*mol/L (95% CI: -19.28, -5.86 *μ*mol/L). The MD of blood phosphorus (*P* = 0.03) was -0.39 mg/dl (95% CI: -0.78, -0.01 mg/dl). The MD of serum uric acid (*P* = 0.004) between the dialysis subgroup and the nondialysis subgroup was -31.58 mmol/L (95% CI: -52.99, -10.17 mmol/L). The mean difference (MD) of IL-6 (*P* = 0.02) in the dialysis subgroup was -1.16 *μ*mol/L (95% CI: -2.16, -0.16 *μ*mol/L). However, there was no significant change of RS on hs-CRP, serum creatinine, blood urea nitrogen (BUN), blood paracresol sulfate, and blood lipid.

**Conclusions:**

The intake of RS reduced the serum IS, serum phosphorus, IL-6, and uric acid levels significantly in dialysis patients, while hs-CRP, serum creatinine, BUN, serum paracresol sulfate, and blood lipid showed no significant changes.

## 1. Introduction

Chronic kidney disease CKD has become a main cause of morbidity and mortality of kidney disease worldwide. CKD affects nearly 16% of the adult population and consumes a disproportionate share of health care resources in both developed and developing countries [1–3]. Patients with CKD experience continuous oxidative stress and inflammation. These conditions are associated with the progression of kidney disease and other complications associated with cardiovascular disease [4]. Recently, some researchers have found that the imbalance of the microbiome that occupies the human gut can be considered a new cardiovascular risk factor in patients with CKD because it is directly associated with inflammation and oxidative stress. With the imbalance of intestinal flora, the structure of intestinal epithelial barrier is destroyed, and the permeability of colon is increased. Some metabolites of bacteria, including uremic toxins (such as IS and precursor of cresol sulfate), enter the blood stream [5, 6]. High levels of sulfate indole of phenol and cresol sulfate are associated with poor prognosis in patients receiving hemodialysis [7].

Resistant starch (RS) is the sum of starch and its degradation products that have not been absorbed by the small intestine. It is defined as a component that is resistant to the hydrolysis of pancreatic amylase in the small intestine and reaches the large intestine. As a dietary fiber, RS can serve as a substrate for microbial fermentation [8, 9]. In addition, some intestinal bacteria promote the fermentation of soluble fiber and RS to produce short chain fatty acids (SCFA). The main function of SCFA is to improve the integrity of the intestinal epithelial barrier and relieve local and systemic inflammation. Otherwise, the increases of SCFA production can decrease the intestinal pH [10–12]. So, the fermentable fiber in patients with chronic kidney disease (CKD) has attracted researchers' interest. An analysis of data from 14,543 participants of the U.S. National Health and Nutrition Examination Survey (III) showed that high dietary fermentable fiber intake was associated with a reduced risk of inflammation and death from kidney disease [13]. A recent systematic review and meta-analysis of 143 CKD participants showed that dietary fiber can reduce BUN and creatinine concentrations and has dose-dependent effects on serum creatinine [14]. Recently, RS has gained attention, and a number of trials have investigated the relationship between RS and serum uremia toxins as well as systemic inflammation and oxidative stress in CKD patients [15–22], but these findings are controversial, for example, Tayebi et al. found that in maintenance hemodialysis patients, a diet rich in RS significantly reduced serum concentrations of paracresol, while there was no significant change in IS levels in the treatment group [20]. Marta et al. found that the plasma level of IS significantly decreased after the addition of RS, and the plasma level of cresol sulfate was not affected [18]. As described above, studies on evaluating the effects of RS in the management of CKD have demonstrated controversial findings, and these results were few systematically reviewed. Therefore, this meta-analysis and systematic review aimed at investigating the effects of resistant starch intake on CKD patients.

## 2. Methods

### 2.1. Search Strategy

A comprehensive search of MEDLINE, Embase, Web of Science, and Cochrane systematic review databases was conducted in July 2020, and all new trials were updated in August 2021. Randomized trials were collected to assess the effects of resistant starch on CKD patients. The retrieval strategy used is as follows: take MEDLINE as an example and set the following retrieval formula: ((high-amylose maize type 2-resistant starch, maize [Mesh] OR Resistant starch OR HAM-RS2 OR Hi-maize 260)) AND (Renal Insufficiency, Chronic [Mesh] OR chronic renal insufficiencies OR chronic kidney insufficiencies OR chronic kidney diseases OR chronic renal diseases OR diabetic nephropathies OR diabetic kidney diseases OR kidney failure OR chronic kidney failure OR kidney disease OR uremia OR dialysis OR continuous ambulatory peritoneal dialysis OR hemodialysis OR renal replacement therapy OR peritoneal dialysis OR Equilibrium dialysis OR extended daily dialysis). The articles are filtered using filters (sensitive search strategies used to ensure the best collection of RCTs in electronic searches). In our study, only randomized controlled trials (RCTs) were identified for inclusion and without any language restriction. In the first step of the retrieval process, the titles and abstracts of each article are carefully filtered to include them. Then, all potentially relevant articles were carefully checked for full text for further identification. References to reviews of the effects of RS on CKD patients were also carefully reviewed for inclusion in potential trials.

### 2.2. Selection Criteria

1. The study was a crossover or parallel designed RCT test, and the duration time was ≥4 weeks
2. To study the effect of resistant starch on CKD patients (including dialysis patients)
3. Adult patients with renal insufficiency or renal failure (over 18 years of age) were eligible for inclusion in the study
4. The study did not address other factors that may have potentially positive or negative effects on kidney function

The main exclusion criteria are as follows:

1. Participants under the age of 18
2. Literature on other results, such as pharmacokinetics and basic research
3. Kidney transplant patients
4. Reviews and editorials

### 2.3. Data Extraction and Quality Assessment

Data were collected independently from each included RCT to extract the mean and standard deviation of the primary and secondary outcome indicators. If the published clinical trial report only reported the median, scope, and size of the trial, the mean and standard deviation were estimated by statistical methods [23], and all differences were resolved by consensus. For these included trials, a data extraction table was used to collect the following data: the first author's name, year of publication, number of participants, characteristics of participants (age, gender, dialysis patient, etc.), intervention and control measures, research design, and content and duration of resistant starch (Table 1). The main outcome indicators were serum IS, serum paracresol sulfate, serum creatinine, and serum urea nitrogen, and the secondary outcome indicators were uric acid level, serum phosphorus, hs-CRP, blood lipid, and IL-6.

Methodological quality and risk of bias of each included RCTs were examined carefully using the method described by the Cochrane Collaboration [24]. The items were as follows: (1) random sequence generation, (2) allocation concealment, (3) blinding of participants and personnel, (4) blinding of outcome assessment, (5) incomplete outcome data, (6) selective reporting, and (7) other sources of bias. All seven items were classified as “low risk of bias,” “high risk of bias,” or “unclear risk of bias.”

### 2.4. Statistical Analyses

Data from each of the included trials were analyzed using the RevMan version 5.3, Copenhagen: Cochrane center for northern Europe, Cochrane Collaboration, 2017. The treatment effect was expressed as the mean difference between the change and its 95% confidence interval (CI), and the summary effect was calculated by assigning a weight to the reciprocal of each trial variance. We also performed a subgroup analysis to investigate the potential effects of CKD type (hemodialysis or nonhemodialysis) on the outcome. Heterogeneity of treatment is as follows: using the *χ*^2^ test and the *I*^2^ test to assess inconsistencies, we used a random-effects model to calculate the pooled effect size. Use of a random-effects model is less likely to produce significant results for pooled effect sizes than use of a fixed-effects model [25]. The *P* value threshold of statistical significance was set at 0.05, and *P* ≤ 0.05 was considered significant and statistically significant.

## 3. Results

### 3.1. Trial Flow/Flow of Included Studies

**Figure 1:**
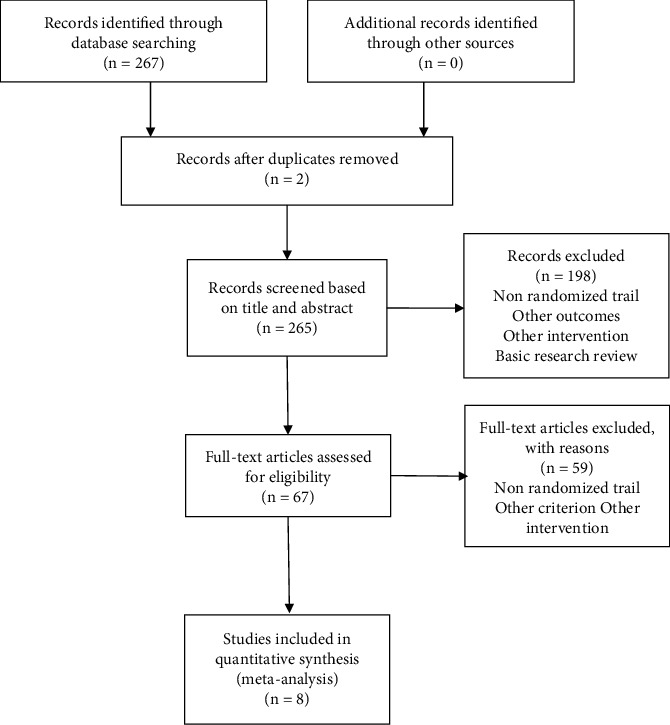
The flow chart of the literature selection process and the reasons for exclusion.

A search of MEDLINE, Embase, Web of Science, and Cochrane systematic review databases identified 267 trials, of which 200 were excluded at the initial screening. Fifty-nine potentially relevant trials were identified for further review, of which eight met our inclusion criteria. All enrolled studies were randomized controlled trials to evaluate the role of RS in patients with CKD. The detailed process of our study selection is shown in Figure 1.

### 3.2. Study Characteristics

Eight studies (301 participants) were identified to assess the impact of RS on CKD patients. Table 1 lists the specific characteristics of the 8 studies. Since Marta et al. 2020 [22] and Andrade et al. 2021 [26] adopted the treatment of intervention—washout period—reintervention, it was divided into two research groups according to the treatment before and after the washout period. According to the type of CKD (dialysis or nondialysis), we divided the data into different subgroups for combined analysis. Seven of the included studies compared RS and common starch. Among the included studies, 7 articles were targeted at dialysis patients. And only one article was nondialysis patients, which is the study object of Meng et al. 2019 [19] which was patients with early type 2 diabetes. As for follow-up time, the duration of these included studies ranged from 4 weeks to 12 weeks.

### 3.3. Quality of Included Studies

**Figure 2:**
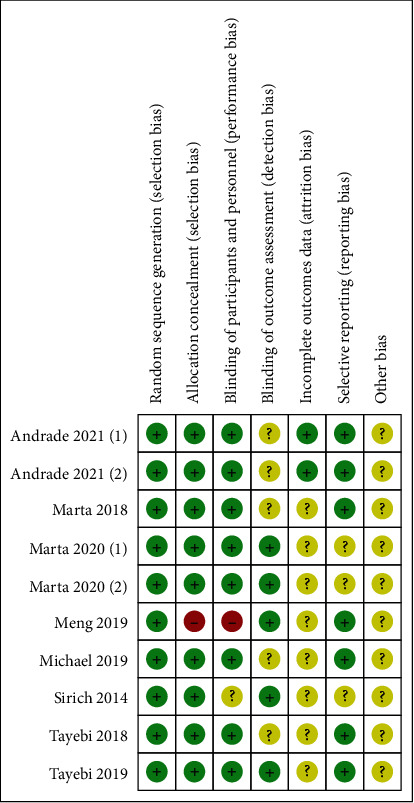
Quality evaluation of included studies and risk of bias summary.

The quality of the included studies varied according to the standard methodology recommended by the Cochrane Collaboration for assessing the risk of bias in Figure 2.

### 3.4. Quantitative Data Synthesis

#### 3.4.1. Indolephenol Sulfate

**Figure 3:**
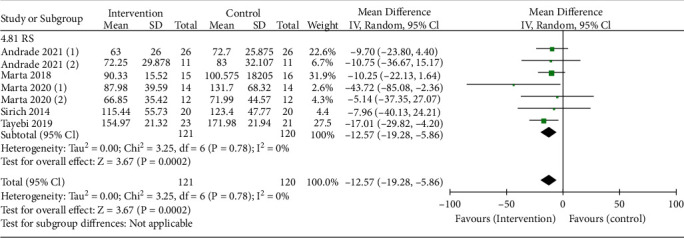
Pooled estimated effect of RS intake on IS (*μ*mol/L) in patients with CKD, with estimated MD and 95% CIs.

A total of five trials were conducted to evaluate the effect of RS intake on CKD. For dialysis patients treated with RS, blood IS was reduced in the RS group compared with the control group, which has statistically significant, and the estimated change of combined effect was -12.57 *μ*mol/L ((95% CI: -19.28, -5.86 *μ*mol/L), *P* = 0.0002), using the random-effects model (Figure 3). In addition, seven trials were insufficient to detect publication bias.

#### 3.4.2. Paracresol Sulfate

**Figure 4:**
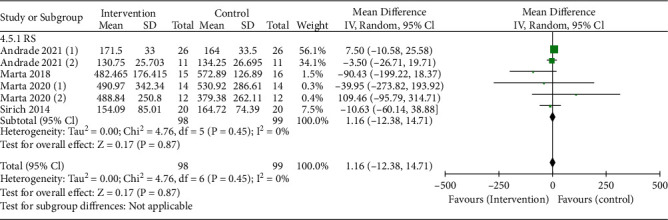
Pooled estimated effect of RS intake on paracresol sulfate (*μ*mol/L) in patients with CKD, with estimated MD and 95% CIs.

A total of four trials were conducted to evaluate the effect of RS intake on CKD. Compared with the control group, there was no significant difference in the change of serum p-cresol sulfate in the RS subgroup, and the combined estimated change was 1.16 *μ*mol/L ((95% CI: -12.38, 14.71 *μ*mol/L), *P* = 0.87) (Figure 4). Using the random-effects model, six trials were insufficient to detect publication bias.

#### 3.4.3. Blood Uric Acid

**Figure 5:**
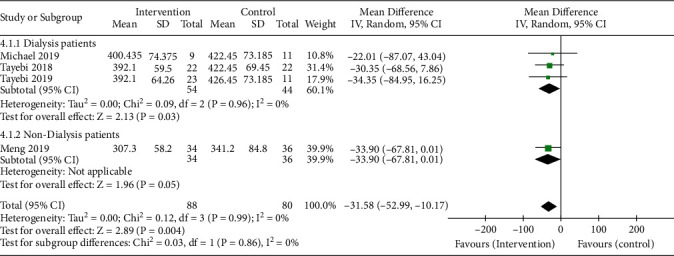
Pooled estimated effect of RS intake on blood uric acid (mmol/L) in patients with CKD, with estimated MD and 95% CIs.

A total of four trials were included, which were divided into two subgroups according to whether they received dialysis or not. Compared with the control group, the serum uric acid level of the dialysis subgroup after RS treatment was significantly reduced, and the combined estimated change was -30.04 mmol/L ((95% CI: -57.65, -2.43 mmol/L), *P* = 0.03). The nondialysis subgroup included only one trial in which the serum uric acid level was reduced, with an estimated change of -33.90 mmol/L ((95% CI: -67.81, 0.01 mmol/L), *P* = 0.05). The total estimated change was -31.58 mmol/L ((95% CI: -52.99, -10.17 mmol/L), *P* = 0.004) (Figure 5). The above random-effects model was used, and four experiments were not enough to detect publication bias.

#### 3.4.4. Blood Phosphorus

**Figure 6:**
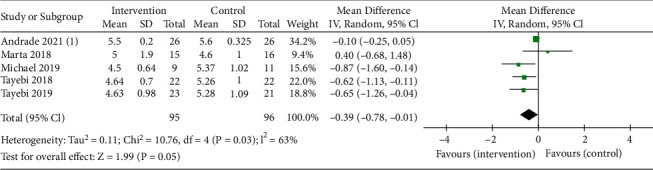
Pooled estimated effect of RS intake on blood phosphorus (mg/dl) in patients with CKD, with estimated MD and 95% CIs.

There were 4 trials, all of which were dialysis combined with RS. Compared with the control group, the blood phosphorus level significantly decreased after treatment, which was statistically significant. The estimated change of the combination was -0.39 mg/dl ((95% CI: -0.78, -0.01 mg/dl), *P* = 0.05) (Figure 6). Using the random-effects model, five trials were insufficient to detect publication bias.

#### 3.4.5. Blood Urea Nitrogen

Five tests were identified to assess the impact of RS intake on CKD, and they were divided into two subgroups according to whether or not they received dialysis. The dialysis and RS subgroups included four tests. Compared with the control group, there was no significant difference in blood urea nitrogen change, and the combined estimated change was -4.94 mg/dl ((95% CI: -10.81, 0.93 mg/dl), *P* = 0.10). Only one trial was included in the nondialysis subgroup, in which there was no significant difference in blood urea nitrogen between the nondialysis and RS subgroups, and the combined estimated change was -0.28 mg/dl ((95% CI: -1.86, 1.30 mg/dl), *P* = 0.73) (Table 2). Using the random-effects model, five trials were insufficient to detect publication bias.

#### 3.4.6. Serum Creatinine

Four tests were determined to evaluate the effect of RS intake on CKD. The patients were divided into two subgroups according to whether they received dialysis or not. Compared with the control group, there was no significant difference in serum creatinine changes between the dialysis and RS subgroups, and the combined estimated changes were -44.94 *μ*mol/L ((95% CI: -171.99, 82.12 *μ*mol/L), *P* = 0.69). The nondialysis subgroup included only one test, in which there was no significant difference in serum creatinine between the nondialysis and RS subgroups, with an estimated change of 4.0 *μ*mol/L ((95% CI: -3.49, 11.49 *μ*mol/L), *P* = 0.30) (Table 2) using the random-effects model. In addition, 3 trials were insufficient to detect publication bias.

#### 3.4.7. hs-CRP

This result was reported in only four trials. No significant difference was observed in the change of hs-CRP after the RS intervention compared with the control, with the combined estimated change of -0.05 mg/dl ((95% CI: -0.15, 0.05 mg/dl), *P* = 0.31) (Table 2). Using the random-effects model, four trials were insufficient to detect publication bias.

#### 3.4.8. IL-6

There were four trials, which were divided into two subgroups according to whether they received dialysis or not. Compared with the control group, there was significant change in IL-6 levels after RS treatment in the dialysis subgroup, and the combined estimated change was -1.16 pg/mL ((95% CI: -2.16, -0.16 pg/mL), *P* = 0.02). There was only one trial in the nondialysis subgroup, in which there was no significant change in blood IL-6 level, with an estimated change of 0.30 pg/mL ((95% CI: -0.17, 0.77 pg/mL), *P* = 0.21). The total estimated change was -0.77 pg/mL ((95% CI: -1.75, 0.21 pg/mL), *P* = 0.12) (Table 2). Using the random-effects model, four trials were insufficient to detect publication bias.

#### 3.4.9. Blood Lipids

This result was reported in 3 trials, which were divided into two subgroups according to whether they received dialysis or not. Compared with the control group, there was no significant change in lipid level after RS treatment in the dialysis subgroup, and the combined estimated change was total cholesterol -0.29 mmol/L ((95% CI: -0.14, 0.72 mmol/L), *P* = 0.19), triglyceride 0.10 mmol/L ((95% CI: -0.25, 0.46 mmol/L); *P* = 0.57), and high-density lipoprotein 0.02 mmol/L ((95% CI: -0.11, 0.15 mmol/L); *P* = 0.76). The nondialysis subgroup included a trial, in which there was no significant change in lipid level, and the change was estimated to be 0.10 mmol/L of total cholesterol ((95% CI: -0.35, 0.55 mmol/L), *P* = 0.21), triglyceride 0.00 mmol/L ((95% CI: -0.33, 0.33 mmol/L; *P* = 1), and high-density lipoprotein 0.10 mmol/L ((95% CI: -0.02, 0.22 mmol/L); *P* = 0.1) (Table 2). Using the random-effects model, 3 trials were insufficient to detect publication bias.

## 4. Discussion

Nutritionists and clinicians have long explored whether different dietary interventions can control or improve the CKD. A recent systematic review and meta-analysis of 143 CKD participants showed that dietary fiber can reduce BUN and creatinine concentrations and has dose-dependent effects on serum creatinine [14]. However, there is an obvious lack of tests to assess other uremia retention solute (such as IS and paracresol sulfate) and inflammatory indicators and only one Meijers' study [27]. In addition, the effects of different fiber types on the CKD were not discussed separately. Recently, RS have gained attention, and a number of trials have investigated the relationship between RS and serum uremia toxins as well as systemic inflammation and oxidative stress in CKD patients [15–22]. As these findings are controversial, further analysis is needed. For example, Tayebi et al. found that in maintenance hemodialysis patients, a diet rich in RS significantly reduced serum concentrations of paracresol, while there was no significant change in IS levels in the treatment group [20]. Marta et al. found that the plasma level of IS significantly decreased after the addition of resistant starch, and the plasma level of cresol sulfate was not affected [18]. Meijers et al. supplemented the diet of hemodialysis patients with inulin in the form of a fructose-rich inulin. P-cresol sulfate production and plasma levels were reduced by 20%, but there was no effect on IS [27].

Some studies have suggested that RS and other dietary fiber components have beneficial effects on patients with CKD, which may involve multiple mechanisms. One is that an RS-rich diet can promote the growth of SCFA-producing bacteria. According to the study of Laffin et al., Faecalibacterium diversity in feces of patients with end-stage renal disease increased significantly after supplementing ham-RS2. The diversity of Faecalibacterium is related to disease state and is also the main bacterium producing butyric acid, whose content is lower in western people, enteritis, and obese people [21]. Vaziri also proved this by finding that diets rich in RS and other fermentable fibers can promote the production of short-chain fatty acids, and the increased production of short-chain fatty acids leads to the reduction of intestinal pH, thus reducing the formation of proinflammatory and prooxidative uremia toxins in the colon [10–12]. SCFA can enable beneficial microorganisms to reproduce and survive, prevent the entry and adhesion of opportunistic bacteria, and reduce the accumulation of toxic substances, thus maintaining the integrity of intestinal epithelium [10–12]. The shortening of colon transport time may also be one of the mechanisms by which RS plays a role. In people with constipation, stool accumulates in the intestines for a long time, and the fermentation of proteins in the intestines leads to the production of toxins. RS can trap water in the intestine, prevent dry stool, help prevent constipation, and reduce fluid overload [10, 27]. In animal experiments, CKD creatinine clearance was significantly improved compared with rats fed the RS diet, with decreased oilfield fibrosis and inflammation, renal tubular damage and reduced NF-KB activation, and increased antioxidant enzyme production, whereas experimental models on the low-fiber diet showed opposite effects [10]. Many studies have shown that metabolic syndrome is closely related to overall health and chronic diseases such as cardiovascular disease, diabetes, and chronic kidney disease. By ingestion of resistant starch, cholesterol and triglycerides are reduced, and insulin sensitivity is improved, which can greatly reduce the incidence of metabolic syndrome. Patients with CKD may also benefit from better glucose metabolism, lipid levels, and better weight management.

As mentioned above, CKD patients have an imbalance of intestinal flora, resulting in changes in intestinal permeability. Some byproducts produced by bacteria metabolizing aromatic amino acids (e.g., tryptophan and tyrosine) include uremia toxins (e.g., IS and precursors of para-cresol sulfate) that enter the blood [5, 6]. CKD patients have high levels of uremia toxins, both because of their increased production and because the damaged kidney cannot be cleared from the bloodstream by urine [28] and because of their low binding rate to plasma proteins and low dialysis clearance [29, 30]. Through the analysis of seven included studies, we found that the intake of RS significantly reduced serum IS in dialysis patients, but there was no significant change in serum p-cresol sulfate. Sirich et al. proposed possible reasons for this inconsistency. In his study, the free fractions of IS and paracresol sulfate in the RS group showed a decreasing trend compared with the control group, while the total solute level decreased less than the free solute level [15]. Since the indices of IS and paracresol sulfate in the included literature were both measured at the total solute level, the free solute level may be a better measurement index, which, of course, needs to be proved by more long-term large-scale RCTS in the future.

Resistant starch also has beneficial effects on the intestinal environment, including increased Ruminococcus bromide. Ruminococcus brucei, one of the main members of Firmicutes, is a major resistant starch fermentation strain. Through its special activity against resistant starch, Ruminococcus brucei releases energy from starch to evade digestion by host enzymes. In addition, the intake of foods rich in resistant starch has been shown to increase intestinal short-chain fatty acid levels, regulate microbial metabolites, and improve glucose homeostasis and insulin sensitivity. Interestingly, the increase in butyric acid levels after taking resistant starch depends on each person's unique gut flora. The effects of resistant starch on intestinal environment indicate that resistant starch has positive effects on the physiological function of intestinal flora, including metabolic activity, nutritional effect on intestinal epithelial and immune structure and function, and protection of host from pathogen invasion.

In this meta-analysis, we found a significant reduction in serum phosphorus concentration, which may be clinically significant because hyperphosphatemia is a problem that is often difficult to solve in patients with predialysis CKD, and the reduction may be due to reduced phosphate intake and reduced intestinal absorption. Phosphorus from plant sources is not well absorbed because much of it is present as a phytic acid and cannot be absorbed well [31].

We also observed a significant reduction in uric acid levels in the RS group. Some studies have shown that dietary fiber can reduce serum uric acid levels by reducing dietary adenine absorption [32, 33].

Some studies have reported that the RS can decrease the serum creatinine and blood urea nitrogen, such as Tayebi [20], but in our meta-analysis, serum creatinine and urea nitrogen levels were no obvious changes, and the duration of the possible reason is that it is included in the study which is too short range (4 weeks to 12 weeks), not very obvious changes in serum creatinine and blood urea nitrogen, and needs more long-term RCT to clarify in the future.

In addition, increased uremia toxins in the blood have been shown to exacerbate inflammation and oxidative stress, as well as endothelial dysfunction and atherosclerotic processes [33–35]. Marta et al. found in their study that the average mRNA Nrf2 expression increased after RS treatment, and Nrf2 is considered to be one of the most important factors for cell defense against oxidative stress and inflammation, demonstrating that cookies rich in resistant starch may reduce the level of indoles sulfate from intestinal flora and reduce inflammation in hemodialysis patients [18]. Tayebi et al. also demonstrated significant reductions in serum TNF-a, IL-6, and malondialdehyde levels in the RS group [17]; however, in our meta-analysis, IL-6 had significant changes in the dialysis group, and there was no significant change in some inflammatory indicators such as hs-CRP in the RS group. One possible reason is still the short duration of the study included, and RS is a longer term process to improve inflammatory markers. Although no significant changes in CRP and serum paracresol sulfate were detected, many articles have shown the RS can reduce inflammation index and dialysis patients' formation of oxidizing uremic toxins. This needs to be elucidated by future large long-term RCTS, too.

Fermentation of the fibers in the colon produces gases including hydrogen and methane, which can cause flatulence and abdominal discomfort. The potential advantage of resistant starch over fructose-rich inulin and other oligosaccharides is that the slow fermentation due to its high molecular weight limits flatulence and other gastrointestinal side effects, and the consumption of RS is well tolerated and significantly ameliorates the prevalence of constipation in CKD patients [17, 27, 34–36].

There are some limitations in our research. First, most of the clinical trials identified with the meta-analysis were relatively short duration and were involved a small number of patients, and the number of trials selected in the meta-analysis for relevant indicators was small; so, changes in aggregate estimates may have been influenced by some studies, particularly those with higher weights. Second, although most of our results showed relatively little or no heterogeneity between studies, there were some differences in the type and amount of RS products used in the intervention and significant differences in the control diet regimens in each study, which may have an impact on the results. Finally, the system review included a crossover study whose data could affect the accuracy of the results because of the washout period. Despite these limitations, based on our meta-analysis, we believe that RS is beneficial to CKD patients, especially hemodialysis patients.

## 5. Conclusions

This meta-analysis indicates that RS reduced the serum IS, serum phosphorus, and uric acid levels significantly in dialysis patients, while hs-CRP, serum creatinine, BUN, serum paracresol sulfate, and blood lipid showed no significant changes.

## Figures and Tables

**Table 1:** Basic characteristics of the included research.

Study, year	Participant	M/F^a^	Control	Intervention	Mean age (S.D. or range), years	Design	Fiber dose (S.D. or range) (g/day)	Duration (weeks)
Sirich et al. 2014 [15]	40 HD^b^	24/16	Waxy corn starch (*N* = 20)	RS (Hi-maize 260) (*N* = 20)	60.5 ± 20.5	R^e^.DB^f^.P^g^	15	6
Tayebi et al., 2018 [17]	44 HD	28/16	Regular wheat flour (*N* = 22)	HAM-RS2 (*N* = 22)	57 ± 16	R.DB.P	20 (0-4 w) 25 (4-8 w)	8
Marta et al., 2018 [18]	31 HD	18/13	Manioc flour (*N* = 16)	Hi-Maize ® 260 (*N* = 15)	53.5 ± 11.5	R.DB.P	16	4
Meng et al., 2019 [19]	70 type 2 DN^c^	39/31	Ordinary staple food (*N* = 36)	High-RS, low-protein flour (*N* = 34)	53.5 ± 11.5	R.DB.P	16	4
Tayebi et al., 2019 [20]	44 HD	N^d^	Waxy corn starch placebo cookie (*N* = 21)	(HAM-RS2) (*N* = 23)	57.90 ± 13.34	R.DB.P	20 (0-4 w) 25 (4-8 w)	8
Laffin et al., 2019 [21]	20 HD	13/7	Fermentable fiber placebo cookies (*N* = 11)	(HAM-RS2) (*N* = 9)	54.3 ± 12.3	R.DB.P	20 (0-4 w) 25 (4-8 w)	8
Marta et al., 2020 (1) [22]	26 HD	N	Manioc flour (*N* = 14)	Cookies rich in resistant starch (*N* = 14)	53.5 ± 11.4	R.TB^h^.C^i^	16	4
Marta et al., 2020 (2) [22]	26 HD	N	Manioc flour (*N* = 12)	Cookies rich in resistant starch (*N* = 12)	53.5 ± 11.4	R.TB.C	16	4
Andrade et al., 2021 (1) [26]	26 PD^h^	14/12	Waxy corn starch (*N* = 26)	Unripe banana flour (RS 48%) (*N* = 26)	55 ± 12	R.TB.C	14	8
Andrade et al., 2021 (2) [26]	11 PD	5/6	Waxy corn starch (*N* = 11)	Unripe banana flour (RS 48%) (*N* = 11)	54 ± 15	R.TB.C	18.7	8

Abbreviations: M/F^a^; ratio of male and female; HD^b^: hemodialysis; DN^c^: diabetic nephropathy; N^d^: none; *R*^e^; randomized; DB^f^: double-blind; TB^h^: triple-blind; C^i^: crossover; P^g^: parallel; PD^h^: peritoneal dialysis.

**Table 2:** Summary of the effects of RS intake in patients with CKD compared with control.

Outcome	No. of studies	No. of populations (intervention/control)	Test for *I*^2^	Heterogeneity (*P*)	Analysis model	Overall effect (*P*)	Mean difference, 95% CI
SCR^a^ (mmol/L)	4	98/99	75%	0.007	Random-effects	0.78	-28.88 (-101.11, 43.35)
HD^b^	3	64/63	77%	0.01	Random-effects	0.69	-44.94 (-171.99, 82.12)
NHD^c^	1	34/36	—	—	—	0.30	4.00(-3.49, 11.49)
hs-CRP^a^ (mg/dl)	4	84/83	1%	0.39	Random-effects	0.31	-0.05(-0.15, 0.05)
IL-6^a^ (pg/mL)	4	84/89	88%	<0.00001	Random-effects	0.12	-0.77 (-1.75, 0.21)
HD^b^	3	50/53	79%	0.008	Random-effects	0.02	-1.16 (-2.16, -0.16)
NHD^c^	1	34/36	—	—	—	0.21	0.30 (-0.17, 0.77)
BUN^a^ (mg/dl)	5	118/119	0%	0.64	Random-effects	0.44	-0.60 (-2.12,0.93)
HD^b^	4	84/83	0%	0.97	Random-effects	0.10	-4.94 (-10.81,0.93)
NHD^c^	1	34/36	—	—	Random-effects	0.73	-0.28 (-1.86,1.30)
TC (mmol/L)^a^	3	79/79	0%	0.82	Random-effects	0.21	0.20 (-0.11, 0.51)
HD^b^	2	45/43	0%	0.86	Random-effects	0.19	-0.29 (-0.14, 0.72)
NHD^c^	1	34/36	—	—	—	0.21	0.10 (-0.35, 0.55)
TG (mmol/L)^a^	3	79/79	0%	0.86	Random-effects	0.70	0.05(-0.20,0.29)
HD^b^	2	45/43	0%	0.72	Random-effects	0.57	0.10 (-0.25,0.46)
NHD^c^	1	34/36	—	—	—	1	0.00 (-0.33, 0.33)
HDL (mmol/L)^a^	3	79/79	0%	0.68	Random-effects	0.16	0.06 (-0.03, 0.15)
HD^b^	2	45/43	0%	1	Random-effects	0.76	0.02 (-0.11, 0.15)
NHD^c^	1	34/36	—	—	—	0.1	0.10 (-0.02, 0.22)

^a^The total effect. HD^b^: hemodialysis subgroup; NHD^c^: nonhemodialysis subgroup; TC: total cholesterol; TG: triglycerides; HDL: high-density lipoprotein; SCR: serum creatinine; BUN: blood urea nitrogen.

## Abbreviations

CKD: Chronic kidney disease
RS: Resistant starch
IS: Indolephenol sulfate
MD: Mean difference
BUN: Blood urea nitrogen
SCFA: Short chain fatty acids
RCTs: Randomized controlled trials.
